# The added value of the cognition, dining, gastrointestinal problems, sleep and tiredness bolt-on dimensions to the EQ-5D-5L in patients with coeliac disease

**DOI:** 10.1007/s10198-024-01719-6

**Published:** 2024-08-30

**Authors:** M. Mercédesz Angyal, Mathieu F. Janssen, Péter L. Lakatos, Valentin Brodszky, Fanni Rencz

**Affiliations:** 1 Károly Rácz Conservative Medicine Division, Semmelweis University Doctoral School, 26 Üllői út, Budapest, H-1085 Hungary; 2 Department of Health Policy, Corvinus University of Budapest, 8 Fővám tér, Budapest, 1093 Hungary; 3 Section Medical Psychology and Psychotherapy, Department of Psychiatry, Erasmus MC, Rotterdam, The Netherlands; 4 McGill University Health Centre, Montreal General Hospital, 1650 Ave. Cedar, D16.173.1, Montreal, QC H3G 1A4 Canada; 5 Department of Internal Medicine and Oncology, Semmelweis University, 2/a Korányi Sándor utca, Budapest, 1083 Hungary

**Keywords:** Coeliac disease, EQ-5D, Bolt-ons, Health-related quality of life, Psychometric properties

## Abstract

**Objectives:**

Multiple studies suggest that the EQ-5D may overestimate health-related quality of life (HRQoL) in patients with coeliac disease (CD). We aimed to develop and psychometrically test potentially relevant bolt-on dimensions to improve the measurement performance of the EQ-5D-5L in CD patients.

**Methods:**

The development and selection of bolt-ons were informed by a literature review on HRQoL in CD, expert and patient input. A cross-sectional online survey was conducted amongst 312 adult CD patients. Respondents completed the EQ-5D-5L, two condition-specific bolt-ons newly-developed for the present study [dining (DI) and gastrointestinal problems (GI)] and three existing bolt-ons [cognition (CO), sleep (SL) and tiredness (TI)]. The following psychometric properties were tested: ceiling, informativity, convergent and known-group validity, and dimensionality (confirmatory factor analysis).

**Results:**

Adding the TI, SL, GI, DI and CO individual bolt-ons reduced the ceiling of the EQ-5D-5L (39%) to 17%, 23%, 24%, 26% and 37%, respectively. GI excelled with strong convergent validity with the Gastrointestinal Symptom Rating Scale total score (r_s_=0.71) and improved the discriminatory power for all known-groups. GI was the only bolt-on loading on a different factor from the five core dimensions, whereas the other four bolt-ons loaded onto the same ‘psychosocial health’ factor as the EQ-5D-5L anxiety/depression dimension.

**Conclusion:**

The DI, GI, SL and TI bolt-ons, especially the GI, enhance the validity of EQ-5D-5L in patients with CD, suggesting their value in capturing important HRQoL aspects potentially missed by the five core dimensions. These bolt-ons can be used in sensitivity analyses supporting health technology assessments and subsequent resource allocation decisions.

**Supplementary Information:**

The online version contains supplementary material available at 10.1007/s10198-024-01719-6.

## Introduction

Generic preference-accompanied measures of health-related quality of life (HRQoL) are widely used in economic evaluations to assess the impact of health conditions and their treatments on HRQoL [1]. The advantages of these measures include the ability to capture the impact of conditions or treatment on the overall HRQoL rather than focusing solely on specific symptoms, and facilitating comparisons across different conditions and disease areas [1]. Among various generic preference-accompanied measures, the EQ-5D is the most commonly used instrument [2]. It has shown good validity and responsiveness in a wide array of acute and chronic health conditions [3]. Moreover, it is recommended by health technology assessment (HTA) guidelines in more than 20 countries [4, 5]. However, the EQ-5D may not perform adequately in all health conditions, given its limitations in content validity in certain HRQoL areas [6].

An area, where the EQ-5D might not fully capture all aspects of HRQoL, is gastrointestinal-related conditions. Although the ‘discomfort’ component of the pain/discomfort composite dimension is intended to assess physical discomfort, potentially incorporating various gastrointestinal symptoms, respondents often use this dimension primarily to report pain, resulting in an underreporting of physical discomfort [7]. Therefore, in any health condition where gastrointestinal problems are relevant, there is a potential to improve the instrument’s validity and sensitivity. An example is coeliac disease (CD), an immune-mediated systemic disorder activated by the ingestion of gluten in genetically susceptible individuals, with a global prevalence of 0.7–1.4% [8–10]. CD patients may struggle not only with gastrointestinal symptoms (e.g. abdominal pain, bloating, diarrhoea, constipation), but also with extra-intestinal symptoms (e.g. fatigue, cognitive impairment, depression) [11–14]. Currently, the only treatment option is a strict, life-long gluten-free diet (GFD) that has a complex impact on the HRQoL of patients [15]. This is due to the demanding nature of the GFD, implying increased dietary costs [16], reduced nutritional value [17], and social constraints [18–20]. The literature suggests that while patients’ HRQoL improves following the GFD, it does not normalize [21].

The EQ-5D has been found to potentially overestimate HRQoL among patients with CD, which is supported by three earlier EQ-5D studies from the UK, Poland and Slovenia reporting better HRQoL in CD patients after diagnosis compared to the general population [22–24]. One possible solution to enhance the coverage of the EQ-5D is the addition of extra dimensions, called ‘bolt-ons’, to the instrument. These additional dimensions may cover specific health problems or dysfunctions relevant to particular conditions or populations [25, 26]. So far, several bolt-on dimensions have been developed for the EQ-5D; for example, a skin irritation and self-confidence bolt-on for patients with chronic skin diseases [27–29], a vision bolt-on for patients with vision problems, such as cataract [30, 31], and a respiratory bolt-on for patients with asthma and chronic obstructive pulmonary disease [32]. The value added by these bolt-ons varies, and their impact on utilities may be limited in some cases [26]. For example, a sleep ‘bolt-on’ dimension to EQ-5D-3L was found to have minimal impact on utilities [33].

This study aimed to develop and assess the psychometric properties of potentially relevant bolt-ons for the EQ-5D-5L in patients with CD. We hypothesized that adding bolt-ons to the EQ-5D-5L would improve the measurement properties, compared to the original EQ-5D-5L.

## Methods

### Development and selection of bolt-ons

In this study, we used both newly developed condition-specific bolt-ons and relevant existing bolt-ons for the EQ-5D-5L (Online Resource S1). The development and selection of bolt-ons were informed by a literature review on HRQoL in CD as well as relevant domains from existing relevant condition-specific measures, and expert input. The panel of experts comprised a CD patient, a gastroenterologist professor and two health economists experienced in utility assessment.

A conceptual model summarising the most important health outcomes in patients with CD has recently been published [34]. This conceptual model, based on an analysis of the item content of condition-specific measures and additional interviews with both CD clinical experts and payers, encompasses two large symptom groups (gastrointestinal and extra-intestinal) and six aspects of HRQoL. The most common gastrointestinal symptoms included in CD-specific measures are bloating, nausea, diarrhoea, abdominal pain/discomfort, loose stool and flatulence. The most common extra-intestinal symptoms in these instruments are low energy/fatigue, headaches, food cravings and slowness/difficulty thinking. The six HRQoL areas include daily activities (e.g. mobility, self-care, reduced concentration), psychological impact (e.g. anxiety, depression, stress, mental fatigue), relationships (e.g. stigmatization, family life), social or leisure (social activities, dining out at restaurants), sleep (e.g. insomnia), and treatment/dietary (e.g. bathroom usage, difficulty adhering to a GFD). While the five dimensions of the EQ-5D-5L seem to provide a good coverage of the main symptoms and HRQoL aspects of CD, there might be some important areas missed out, where the addition of bolt-ons could be particularly useful. Based on the frequency of the abovementioned symptoms and HRQoL impacts and discussions between members of the expert panel, the decision was taken to develop two new condition-specific bolt-on items to more comprehensively reflect the psychosocial and physical burden of CD on HRQoL: dining (DI), which featured examples of ‘following a diet’ and ‘eating out’, and gastrointestinal problems (GI), listing the following examples in parentheses: diarrhoea, constipation, nausea, vomiting, heartburn, bloating and gases. Furthermore, we selected three existing bolt-ons that held relevance in the context of CD: cognition (CO), sleep (SL) and tiredness (TI) [35, 36], presented in Online Resource S1. The CO bolt-on was deemed especially relevant as CD patients can experience certain neurologic and psychiatric manifestations, including cerebellar ataxia, peripheral neuropathy, brainstem dysfunction, epilepsy, dementia, headache and depression [37–39]. A ‘social relationships’ bolt-on dimension was not included in the study as we considered that it would potentially overlap with some aspects of HRQoL covered by the DI bolt-on (eating out) as well as the usual activities EQ-5D-5L dimension.

In developing the two new bolt-ons, we aimed to formulate the items in a way that could potentially make them suitable also for measuring HRQoL in other health conditions with similar impacts on patients’ lives. For instance, the GI bolt-on could be applied in any disease characterised by gastrointestinal problems, while the DI bolt-on could be used in other diseases requiring specific diets, such as diabetes. Both bolt-on items were framed the same manner as the EQ-5D-5L items, featuring a short dimension title accompanied by a few examples in parentheses and the same number of and severity-type response levels ranging from ‘no problems’ to ‘extreme problems’ [25]. When selecting the examples for the items, we relied on both the language used in qualitative expert interviews in an earlier study and item wordings of commonly used condition-specific instruments [34]. The language and wording of the two bolt-on items were finalized based on input gathered from a CD patient and subsequent discussions within the expert panel.

### Cross-sectional survey among CD patients

An online cross-sectional survey was conducted among 312 Hungarian adult CD patients between December 2020 and January 2021 [40]. Permission for conducting the survey was obtained from the Research Ethics Committee of the Corvinus University of Budapest (reference no. KRH/390/2020). A convenience sample of CD patients was recruited through different patient organisations and social media groups. Participation in the survey was voluntary and anonymous, with no incentives offered. The survey was administered using Qualtrics (Qualtrics 2020, Provo, UT, USA). To be eligible for participation in the study, respondents needed to be aged 18 years or older, provide informed consent and confirm their diagnosis of CD.

The questionnaire consisted of four parts. The first part included questions about CD-related clinical characteristics, adherence to GFD, disease duration, and any comorbidities and symptoms experienced related to CD. The second part comprised various standardised measures to assess HRQoL and wellbeing, while the third part employed preference elicitation methods to evaluate respondents’ current own health and hypothetical health state vignettes. The final section collected sociodemographic data, such as age, sex, place of residence and employment status. All questions were mandatory, and as a result, there were no missing responses.

### Outcome measures

Three main outcome measures were included in the questionnaire: EQ-5D-5L with bolt-ons, Satisfaction with Life Scale (SWLS), and the Gastrointestinal Symptom Rating Scale (GSRS).

The EQ-5D-5L comprises two parts: a five-dimensional descriptive system and a visual analogue scale (EQ VAS) [41]. The descriptive system has five dimensions, each represented by one item: mobility (MO), self-care (SC), usual activities (UA), pain/discomfort (PD), and anxiety/depression (AD). For each item, responses are based on a 5-point severity scale, ranging from ‘no problems’ to ‘extreme problems/unable to’. A total of 5^5^=3125 different health profiles may be described by the five dimensions. For instance, the profile of 11111 indicates that the patient has no problems in any of the five dimensions, representing the best possible health state. While the EQ-5D-5L can be scored using national value sets for the calculation of quality-adjusted life years (QALYs) [42], this approach was not used in this study. Instead, level sum scores (LSSs) were computed by taking an unweighted sum of the responses on each of the five dimensions [43]. The EQ VAS is a vertical hash marked scale with the endpoints of 0 (the worst health you can imagine) and 100 (the best health you can imagine) [41].

In our questionnaire, the five bolt-on items were completed after the EQ VAS in the following order: DI, GI, CO, TI and SL. Each bolt-on was presented on a separate screen. The total number of possible health profiles increases substantially when adding bolt-ons to the EQ-5D-5L. It becomes as high as 5^6^=15,625 when including one, 5^7^=78,125 when including two bolt-ons, and so forth.

CD-specific symptoms were measured with the Gastrointestinal Symptom Rating Scale (GSRS) [44], a widely used instrument in CD patients [45–47]. The GSRS comprises 15 items organised into five domains: reflux (2 items), abdominal pain (3 items), indigestion (4 items), diarrhoea (3 items) and constipation (3 items). Each item has seven response options ranging from ‘no discomfort at all’ (= 1) to ‘very severe discomfort’ (= 7). Adding up the item scores, the total score may range from 15 to 105, where lower scores correspond to fewer health problems.

The Satisfaction with Life Scale (SWLS) was used to assess the patients’ subjective wellbeing [48]. This measure consists of 5 statements, each rated on a 7-point Likert scale. The possible scores range from 5 to 35, where lower scores indicate dissatisfaction, and higher scores denote greater life satisfaction.

### Analyses of psychometric properties

We followed the analytical framework used to test psychometric properties of bolt-ons in multiple previous studies [25, 26, 49]. First, each individual bolt-on was tested separately, and subsequently, various combinations of bolt-ons were examined. The selection of bolt-on combinations was conducted incrementally, considering the performance of each individual bolt-on. Data were analysed using Stata 14.0 (StataCorp. 2015, College Station, TX, USA) and R 4.2.0 (R Core Team, 2022, Vienna, Austria) and SPSS 27.0 (IBM Corp., 2023, Armonk, NY, USA). For all analyses, a *p* < 0.05 was considered statistically significant.

#### Distributional characteristics, ceiling and informativity

Descriptive analysis was used to summarise the demographic and clinical characteristics of the patients. The distributions of patients’ responses across different levels of the EQ-5D-5L and bolt-on items were presented as relative frequencies. We determined the frequency of different health profiles within the sample upon the addition of bolt-on(s). We calculated the ceiling both at the level of individual items and at the level of the instrument itself (i.e. proportion of patients with the best possible health profile in the EQ-5D-5L with and without bolt-on(s)). Similarly we explored the absolute (Shannon index, H’) and relative informativity (Shannon evenness index, J’) of each individual bolt-on and the EQ-5D-5L plus bolt-on(s) [50]. We hypothesized that adding bolt-on(s) would lead to a reduction in the ceiling and an improvement in both the absolute and relative informativity.

#### Convergent and divergent validity

Spearman’s rank-order correlations (r_s_) were used to test the associations between EQ-5D-5L dimensions, bolt-ons, GSRS domains and total score, and EQ VAS. Correlation coefficients were interpreted as very weak (< 0.20), weak (0.20–0.39), moderate (0.40–0.59), strong (0.60–0.79) and very strong (≥ 0.80) [51]. We assumed that the GI bolt-on would demonstrate moderate or strong correlation with GSRS domains and total score as well as with the pain/discomfort EQ-5D-5L dimension. This latter hypothesis arises from the understanding that certain gastrointestinal symptoms are associated with at least some physical discomfort [7]. Further, we anticipated a moderate correlation between (i) CO and TI with usual activities; (ii) SL and TI with pain/discomfort; (iii) CO, SL and TI with anxiety/depression [52–56]. Regarding the DI bolt-on, our hypothesis was that the bolt-on item would exhibit a (very) weak correlation with the five core EQ-5D-5L items due to the conceptual distinction between them.

#### Known-group validity

To evaluate the known-group validity of the EQ-5D-5L with bolt-on(s) as compared to the EQ-5D-5L alone, mean LSSs were computed and subsequently standardized to a 0-100 scale to ensure score comparability. Patients were categorised into known groups based on health status, as assessed on a 5-point scale (poor, fair, good, very good and excellent), tertiles of GSRS total scores and the presence of any symptoms at the time of the survey. To quantify the relative efficiency in detecting differences among these known groups, we calculated the ratio of the F-statistic from the analysis of variance. The EQ-5D-5L was taken as a reference for determining relative efficiency; thus, an F-ratio > 1 indicated that EQ-5D-5L with bolt-on(s) was more efficient at distinguishing across groups. To assess the statistical significance of an F-ratio differing from 1, we estimated 95% confidence intervals using 3000 bootstrap replications. Additional bolt-ons were added to the EQ-5D-5L until the newly included bolt-on resulted in a statistically significant improvement in relative efficiency (higher ANOVA F values), thereby indicating an improvement in the known-group validity of the instrument.

#### Explanatory power

We performed both univariable and multivariable linear regression analyses to compare the exploratory power of the EQ-5D-5L and bolt-on items. In all models, the EQ VAS or SWLS total scores were used as dependent variables. Furthermore, for every case, two different models were run: one unadjusted and one adjusted for age, gender and GSRS score. In the univariable models, we individually examined the five items of the EQ-5D-5L and the five bolt-on items. Multivariable models were developed incrementally by adding bolt-on items to the model, along with the five EQ-5D-5L items, that contributed the most to the adjusted R^2^. If the inclusion of a bolt-on item failed to increase the adjusted R², it was subsequently excluded from the model.

#### Dimensionality analyses

To explore the underlying factor structure of the bolt-ons, we employed principal component analysis (PCA) and confirmatory factor analysis (CFA). We included the five items EQ-5D-5L, the five bolt-ons, the five GSRS domains and the five SWLS items in these analyses. The PCA was performed using a promax rotation, and the number of factors was determined based on the Kaiser’s criterion [57]. Factor loadings were interpreted based on the following reference values: ≤0.32 (unacceptable), 0.33–0.44 (poor), 0.45–0.54 (fair), 0.55–0.62 (good), 0.63–0.70 (very good) and ≥ 0.71 (excellent) [58]. CFA was used to test whether the data fit to our hypothesized measurement model. To address the ordinal nature of items, we employed the diagonally weighted least squares (DWLS) estimator to compute factor loadings. Model fit was considered acceptable when the root mean square error of approximation (RMSEA) was less than 0.08 and the comparative fit index (CFI) exceeded 0.90 [59].

## Results

### Characteristics of the patient population

The mean age was 35.8 (SD 11.5), ranging from 18 to 80 years, and the majority of patients were female (70.2%) (Table 1). Most patients (89.4%) reported to have at least one comorbidity with the most common being allergies, other food intolerances and gastroesophageal reflux disease. Over two-thirds (70.8%) of the patients experienced symptoms of CD in the previous week, with fatigue being the most commonly reported. Among gastrointestinal symptoms, flatulence, heartburn, diarrhoea, upset stomach, constipation and nausea were reported by 25.3%, 22.8%, 18.3%, 12.5%, 10.3% and 5.8%, respectively.

**Table 1:** Characteristics of the patient population

Variables	Mean or *n*	SD or %	Variables	Mean or *n*	SD or %
**Sex**			Most common comorbidities*		
Female	219	70.2%	Allergies	110	35.3%
Male	93	29.8%	Other food intolerance	96	30.8%
**Age (years)**	35.8	11.5	Gastroesophageal reflux disease	86	27.6%
18–24	59	18.9%	Hair loss	67	21.5%
25–34	98	31.4%	Thyroid disease	62	19.9%
35–44	73	23.4%	Iron deficiency	56	17.9%
45–54	68	21.8%	Eczema	44	14.1%
55+	14	4.5%	Hypertension	40	12.8%
**Place of residence**			Depression	37	11.9%
Capital	93	29.8%	Anaemia	34	10.9%
County town	69	22.1%	Rheumatic disease	30	9.6%
Other town	76	24.4%	Inflammatory bowel disease	12	3.8%
Village	74	23.7%	Symptoms in the previous week*		
**Highest level of education**			Fatigue	118	37.8%
Primary school	8	2.6%	Flatulence	79	25.3%
Secondary school	147	47.1%	Headache	76	24.4%
College/university	157	50.3%	Heartburn	71	22.8%
**Employment**			Abdominal pain	71	22.8%
Full-time/self employed	211	67.6%	Diarrhoea	57	18.3%
Student	44	14.1%	Mood swings	53	17.0%
Part-time employed	19	6.1%	Hair loss	51	16.3%
Unemployed	10	3.2%	Joint pain	47	15.1%
Other	28	9.0%	Skin rash	39	12.5%
**Following a gluten-free diet**	312	100%	Upset stomach	39	12.5%
**Age at diagnosis**	27.1	14.0%	Constipation	32	10.3%
**Number of comorbidities**			Depression	20	6.4%
0	33	10.6%	Nausea	18	5.8%
1	74	23.7%	Mouth ulcer	16	5.1%
2–3	101	32.4%	Weight loss	11	3.5%
4+	104	33.3%	Other symptoms	8	2.6%
**Gastrointestinal Symptom Scale (GSRS) score**	28.3	11.7	**No symptoms in the previous week**	91	29.2%

*More than one may occur in each patient

### Distributional characteristics, ceiling and informativity

The distribution of responses to the EQ-5D-5L dimensions and bolt-ons is shown in Table 2. Among the five core dimensions, SC demonstrated the highest ceiling (98.1%), followed by MO (82.7%), UA (77.9%), AD (59.0%), and PD (58.7%) (Table 3). For all but one (CO) of the bolt-ons, the ceiling was lower than that of any of the five core EQ-5D-5L dimensions. Based on the ceiling, the order of the bolt-ons was as follows: TI (22.8%), GI (39.1%), SL (44.6%), DI (52.9%), and CO (74.0%). The ceiling of the EQ-5D-5L was 38.8% (i.e. proportion of 11111 profiles). Adding the TI, SL, GI, DI and CO individual bolt-ons reduced the ceiling to 17.0%, 23.1%, 24.0%, 26.3% and 36.5%, respectively. When adding all five bolt-ons to the EQ-5D-5L, the ceiling was reduced to 7.4%. The number of profiles significantly increased by adding bolt-ons to the EQ-5D-5L, with the largest increase observed with DI, where the number of profiles nearly doubled.

**Table 2:** Responses on the five EQ-5D-5L dimensions and five bolt-ons

Dimensions	No problems		Slight problems		Moderate problems		Severe problems		Extreme problems/ unable to	
	*n*	%	*n*	%	*n*	%	*n*	%	*n*	%
Mobility (MO)	258	82.7%	39	12.5%	12	3.8%	3	1.0%	0	0.0%
Self-care (SC)	306	98.1%	5	1.6%	1	0.3%	0	0.0%	0	0.0%
Usual activities (UA)	243	77.9%	58	18.6%	6	1.9%	5	1.6%	0	0.0%
Pain/discomfort (PD)	183	58.7%	99	31.7%	24	7.7%	5	1.6%	1	0.3%
Anxiety/depression (AD)	184	59.0%	95	30.4%	24	7.7%	6	1.9%	3	1.0%
Cognition (CO)	231	74.0%	65	20.8%	13	4.2%	3	1.0%	0	0.0%
Dining (DI)	165	52.9%	77	24.7%	57	18.3%	11	3.5%	2	0.6%
Gastrointestinal problems (GI)	122	39.1%	120	38.5%	62	19.9%	7	2.2%	1	0.3%
Sleep (SL)	139	44.6%	119	38.1%	39	12.5%	14	4.5%	1	0.3%
Tiredness (TI)	71	22.8%	148	47.4%	75	24.0%	14	4.5%	4	1.3%

**Table 3:** Ceiling, informativity and total number of health profiles on EQ-5D-5L and bolt-ons

**Dimensions**	Ceiling		Ceiling EQ-5D-5L (+bolt-ons)		Absolute ceiling reduction (%)	Relative ceiling reduction (%)	
**EQ-5D-5L**	n	%	n	%			
Mobility (MO)	258	82.7%			-	-	
Self-care (SC)	306	98.1%			-	-	
Usual activities (UA)	243	77.9%	121	38.8%	-	-	
Pain/discomfort (PD)	183	58.7%			-	-	
Anxiety/depression (AD)	184	59.0%			-	-	
**Bolt-ons**							
Dining (DI)	165	52.9%	82	26.3%	-12.5%	32.2%	
Gastrointestinal problems (GI)	122	39.1%	75	24.0%	-14.7%	38.0%	
Cognition (CO)	231	74.0%	114	36.5%	-2.2%	5.8%	
Sleep (SL)	139	44.6%	72	23.1%	-15.7%	40.5%	
Tiredness (TI)	71	22.8%	53	17.0%	-21.8%	56.2%	
**Combinations of bolt-ons**							
GI + TI	45	14.4%	37	11.9%	-26.9%	69.4%	
GI + TI + SL	32	10.3%	28	9.0%	-29.8%	76.9%	
GI + TI + SL + DI	26	8.3%	23	7.4%	-31.4%	81.0%	
All five bolt-ons	26	8.3%	23	7.4%	-31.4%	81.0%	

Dimensions	Item-level informativity		Informativity EQ-5D-5L (+bolt-ons)		Total number of health state profiles		
**EQ-5D-5L**	H'	J'	H'	J'	Observed (n)	%	Theoretical maximum
Mobility (MO)	0.85	0.36					
Self-care (SC)	0.15	0.06					
Usual activities (UA)	0.94	0.40	3.71	0.32	47	1.50%	3125
Pain/discomfort (PD)	1.38	0.60					
Anxiety/depression (AD)	1.43	0.62					
**Bolt-ons**							
Dining (DI)	1.65	0.71	4.95	0.36	90	0.58%	15625
Gastrointestinal problems (GI)	1.67	0.72	4.84	0.35	81	0.52%	15625
Cognition (CO)	1.05	0.45	4.33	0.31	75	0.48%	15625
Sleep (SL)	1.65	0.71	4.94	0.35	85	0.54%	15625
Tiredness (TI)	1.77	0.76	4.88	0.35	80	0.51%	15625
**Combinations of bolt-ons**							
EQ-5D-5L + DI + SL	-	-	5.91	0.36	130	0.17%	78125
EQ-5D-5L + DI + SL + GI	-	-	6.63	0.36	169	0.04%	390625
EQ-5D-5L + DI + SL + GI + TI	-	-	7.18	0.34	208	0.01%	1953125
EQ-5D-5L + 5 bolt-ons	-	-	7.28	0.31	219	0.00%	9765625

All bolt-ons but CO demonstrated both a higher absolute and relative informativity than any of the five core dimensions (Table 3). The TI and GI bolt-on items showed the highest relative informativity. Absolute informativity increased with the addition of any of the five bolt-ons to the EQ-5D-5L (from 3.71 to 4.33–4.95). Moreover, relative informativity also increased with the addition of the bolt-ons, except for CO. The highest improvement in relative informativity was achieved by adding the DI bolt-on. Absolute informativity increased with adding an increasing number of bolt-ons; however, relative informativity increased only up to four bolt-ons.

### Convergent and divergent validity

The correlations between the EQ-5D-5L, EQ VAS, bolt-ons and GSRS domains and total score are shown in Table 4. In line with our hypotheses, a moderate correlation was observed between GI and PD (r_s_=0.508), between TI and PD (r_s_=0.465) and between TI and AD (r_s_=0.425). The GI bolt-on was strongly correlated with GSRS total score (r_s_=0.712) and moderately with each GSRS domain (range of r_s_: 0.419 to 0.584) as expected. The CO, DI and SL bolt-ons exhibited only weak or very weak correlations with any of the five core dimensions of the EQ-5D-5L.

**Table 4:** Convergent and divergent validity of the EQ-5D-5L and five bolt-ons

Variables	EQ-5D-5L					Bolt-ons					EQ VAS	GSRS total score	GSRS Diarrhoea	GSRS Indigestion	GSRS Constipation	GSRS Abdo-minal pain
	Mobility	Self-care	Usual activities	Pain/discomfort	Anxiety/depression	Dining	Gastro-intest. prob.	Cog-nition	Sleep	Tired-ness						
Mobility	-															
Self-care	0.285	-														
Usual activities	0.429	0.300	-													
Pain/discomfort	0.365	0.240	0.499	-												
Anxiety/depression	0.159	0.174	0.273	0.407	-											
Dining	0.163	0.098*	0.333	0.283	0.271	-										
Gastrointestinal problems	0.200	0.103*	0.357	0.508	0.372	0.383	-									
Cognition	0.165	-0.016*	0.391	0.374	0.365	0.326	0.369	-								
Sleep	0.202	0.087*	0.269	0.335	0.294	0.245	0.309	0.314	-							
Tiredness	0.313	0.136	0.390	0.465	0.425	0.355	0.403	0.399	0.376	-						
EQ VAS	-0.329	-0.193	-0.424	-0.542	-0.317	-0.229	-0.504	-0.347	-0.262	-0.388	-					
GSRS total score	0.222	0.139	0.376	0.534	0.344	0.359	0.712	0.331	0.364	0.492	-0.462	-				
GSRS Diarrhoea	0.161	0.071*	0.286	0.370	0.227	0.326	0.467	0.221	0.264	0.304	-0.264	0.650	-			
GSRS Indigestion	0.126	0.069*	0.222	0.342	0.234	0.269	0.565	0.226	0.261	0.426	-0.373	0.815	0.435	-		
GSRS Constipation	0.147	0.102*	0.314	0.365	0.248	0.190	0.419	0.288	0.292	0.250	-0.248	0.660	0.358	0.430	-	
GSRS Abdominal pain	0.193	0.075*	0.386	0.484	0.295	0.350	0.584	0.315	0.291	0.431	-0.403	0.808	0.433	0.567	0.447	-
GSRS Reflux	0.246	0.128	0.312	0.497	0.308	0.231	0.478	0.246	0.359	0.397	-0.358	0.650	0.330	0.356	0.372	0.553

GSRS = Gastrointestinal Symptoms Rating Scale; EQ VAS = EQ Visual Analogue Scale; SWLS = Satisfaction with Life Scale

*p ≥ 0.05

### Known-group validity

The addition of the GI bolt-on significantly improved the ability of the EQ-5D-5L to discriminate between groups of patients based on self-perceived health status, GSRS tertiles and the presence of symptoms, with relative efficiencies ranging between 1.30 (95%CI 1.14–1.49) and 1.84 (95%CI 1.56–2.23) (Table 5). After the inclusion of the GI bolt-on, no additional bolt-ons were able to further improve the known-group validity of the instrument.

**Table 5:** Known groups validity of the EQ-5D-5L plus bolt-ons

	Mean (SD) EQ-5D-5L + bolt-on LSS (0-100)			RE (95%CI), ref: previous row
**Health status**	**Poor-fair**	**Good**	**Very good-excellent**	
n	75	141	96	-
EQ-5D-5L	17.6 (14.25)	6.81 (7.45)	2.45 (4.04)	-
EQ-5D-5L + GI	20.83 (13.44)	9.31 (7.69)	3.56 (4.87)	1.30 (1.14–1.49)
**Gastrointestinal symptoms (GSRS tertiles)** ^***a***^	**< 21**	**22–30**	**30+**	-
n	111	99	102	-
EQ-5D-5L	3.24 (5.59)	6.97 (6.84)	14.36 (13.94)	-
EQ-5D-5L + GI	3.75 (5.36)	9.43 (7.16)	18.30 (13.05)	1.84 (1.56–2.23)
**Symptomatic**	**No symptoms**		**At least one symptom**	
n	90		222	-
EQ-5D-5L	3.00 (7.10)		10.11 (10.99)	-
EQ-5D-5L + GI	3.56 (6.45)		13.04 (11.11)	1.79 (1.49–2.44)

CI = confidence interval; GI = gastrointestinal problems bolt-on; GSRS = Gastrointestinal Symptoms Rating Scale; LSS = level sum score

*a*: Higher scores represent worse gastrointestinal symptoms

### Exploratory power

The results of the univariable linear regression analysis revealed that UA (adjusted R^2^ = 0.304) and PD (adjusted R^2^ = 0.301) demonstrated the highest explanatory power for EQ VAS scores (Table 6). The CO (adjusted R^2^ = 0.196) and GI (adjusted R^2^ = 0.179) bolt-ons performed the best among the bolt-ons. With regard to SWLS, AD (adjusted R^2^ = 0.140) and PD (adjusted R^2^ = 0.116) exhibited the highest explanatory power, while TI (adjusted R^2^ = 0.103) and GI (adjusted R^2^ = 0.095) demonstrated the highest values among the bolt-ons. The addition of bolt-ons to the EQ-5D-5L improved the explained variance of EQ VAS and SWLS scores, as indicated by adjusted R^2^. Specifically, two bolt-ons (GI and CO) improved the explained variance in EQ VAS from 0.411 to 0.440, while three bolt-ons (DI, GI and SL) improved the explained variance in SWLS from 0.193 to 0.206. When adjusting the models for age, gender and GSRS score, there were only minimal differences in the best-performing bolt-ons, and the contributions of CO and GI to explaining health (EQ VAS) and DI to wellbeing (SWLS) were confirmed.

**Table 6:** The explanatory power of EQ-5D-5L and bolt-ons for EQ VAS and SWLS

Dimensions	EQ VAS (unadjusted models)		EQ VAS (models adjusted for age, gender and GSRS)		SWLS (unadjusted models)		SWLS (models adjusted for age, gender and GSRS)	
	Adjusted *R*^2^	ΣΔ adjusted *R*^2^	Adjusted *R*^2^	ΣΔ adjusted *R*^2^	Adjusted *R*^2^	ΣΔ adjusted *R*^2^	Adjusted *R*^2^	ΣΔ adjusted *R*^2^
Individual dimensions								
Mobility (MO)	0.1587	-	0.2632	-	0.0345	-	0.1137	-
Self-care (SC)	0.0886	-	0.2481	-	0.0044	-	0.1040	-
Usual activities (UA)	0.3039	-	0.3456	-	0.1101	-	0.1555	-
Pain/discomfort (PD)	0.3012	-	0.3352	-	0.1156	-	0.1519	-
Anxiety/depression (AD)	0.1826	-	0.2775	-	0.1399	-	0.1873	-
Gastrointestinal problems (GI)	0.1787	-	0.2191	-	0.0954	-	0.1208	-
Cognition (CO)	0.1958	-	0.2995	-	0.0950	-	0.1626	-
Sleep (SL)	0.1085	-	0.2161	-	0.0718	-	0.1300	-
Tiredness (TI)	0.1617	-	0.2369	-	0.1029	-	0.1506	-
Dining (DI)	0.0652	-	0.2033	-	0.0802	-	0.1435	-
**EQ-5D-5L(+ bolt-ons)**								
MO + SC + UA + PD + AD	0.4109	-	0.4209	-	0.1934	-	0.2203	-
MO + SC + UA + PD + AD + CO	0.4293	0.0184	0.4416	0.0206	-	-	-	-
MO + SC + UA + PD + AD + CO + GI	0.4402	0.0293	0.4463	0.0254	-	-	-	-
MO + SC + UA + PD + AD + DI	-	-	-	-	0.2039	0.0105	0.2309	0.0107
MO + SC + UA + PD + AD + DI + SL	-	-	-	-	0.2052	0.0118	-	-
MO + SC + UA + PD + AD + DI + SL + GI	-	-	-	-	0.2057	0.0123	-	-
MO + SC + UA + PD + AD + DI + TI	-	-	-	-	-	-	0.2333	0.0130
MO + SC + UA + PD + AD + DI + TI + CO	-	-	-	-	-	-	0.2334	0.0131

EQ VAS = EQ Visual Analogue Scale; SWLS = Satisfaction with Life Scale; GSRS = Gastrointestinal Symptoms Rating Scale

### Dimensionality

The PCA identified four factors (sorted by descending eigenvalue): ‘gastrointestinal problems’, ‘satisfaction with life’, ‘psychosocial health’ and ‘pain and usual activities’ (Online Resource S2). These factors explained 61.9% of the total variance. The GSRS domains and GI bolt-on loaded onto the ‘gastrointestinal problems’ factor, while the remaining four bolt-ons along with the EQ-5D-5L AD item loaded onto the ‘psychosocial health’ factor. All five SWLS items loaded onto the ‘satisfaction with life’ factor, whereas the ‘pain and usual activities’ factor was constituted by the first four EQ-5D-5L items. The EQ-5D-5L PD item was loaded on both the ‘gastrointestinal problems’ and ‘pain and usual activities’ factors, with a higher factor loading for the latter. The CFA confirmed the results of PCA in terms of the number of factors identified and the relationships between items and factors (Table 7). The model showed an appropriate fit with an RMSEA of 0.064 and CFI of 0.927. All but two items (EQ-5D-5L SC and DI bolt-on) had a standardized factor loading of > 0.55, suggesting a good fit. Only the GI bolt-on loaded on a completely different factor than any EQ-5D-5L item; the other four bolt-ons were loaded on the same ‘psychosocial health’ factor as the EQ-5D-5L AD item.

**Table 7:** Standardized factor loadings in the confirmatory factor analysis

Items	Loadings per factors			
	1. Gastrointestinal problems	2. Satisfaction with life	3. Psychosocial health	4. Pain and usual activities
GSRS Abdominal pain	0.826	-	-	-
EQ-5D-5L Gastrointestinal problems bolt-on	0.762	-	-	-
GSRS Indigestion	0.711	-	-	-
GSRS Reflux	0.684	-	-	-
GSRS Constipation	0.620	-	-	-
GSRS Diarrhoea	0.593	-	-	-
SWLS The conditions of my life are excellent.	-	0.884	-	-
SWLS So far I have gotten the important things I want in life	-	0.851	-	-
SWLS I am satisfied with my life.	-	0.794	-	-
SWLS In most ways my life is close to my ideal.	-	0.792	-	-
SWLS If I could live my life over, I would change almost nothing	-	0.714	-	-
EQ-5D-5L Tiredness bolt-on	-	-	0.711	-
EQ-5D-5L Sleep bolt-on	-	-	0.679	-
EQ-5D-5L Cognition bolt-on	-	-	0.613	-
EQ-5D-5L Anxiety/depression bolt-on	-	-	0.574	-
EQ-5D-5L Dining bolt-on	-	-	0.523	-
EQ-5D-5L Pain/discomfort	-	-	-	0.776
EQ-5D-5L Usual activities	-	-	-	0.773
EQ-5D-5L Mobility	-	-	-	0.590
EQ-5D-5L Self-care	-	-	-	0.454

GSRS = Gastrointestinal Symptoms Rating Scale

SWLS = Satisfaction with Life Scale

## Discussion

This study presented the psychometric testing of two newly-developed (dining and gastrointestinal problems) and three existing (cognition, sleep, tiredness) bolt-on items for the EQ-5D-5L in patients with CD. The addition of any of the five bolt-ons reduced the ceiling and increased the absolute informativity of the instrument; however, the usefulness of CO seemed marginal in this study population. While the SL and TI bolt-ons exhibited strong descriptive characteristics, the GI and DI bolt-ons appeared to perform slightly better in this specific population, which consisted of patients all following a GFD.

The GI bolt-on demonstrated excellent measurement performance, including strong convergent validity with the GSRS domains and total score, enhanced explanatory power for EQ VAS and significantly improved known-group validity. Notably, this was the only bolt-on that loaded on a different factor from any of the five core dimensions, supporting its value in capturing an aspect of HRQoL not addressed by the core dimensions. However, earlier qualitative and quantitative findings indicate that the impact of gastrointestinal problems can, to some extent, be picked up by the pain/discomfort item [7, 60–62]. This aligns with our convergent validity results, showing a moderate correlation (r_s_=0.51) between the GI bolt-on and the pain/discomfort item. Previous research suggests that several respondents use this item mainly to report ‘pain’, but not other forms of physical discomfort [7, 63, 64]. Targeted bolt-ons addressing specific aspects of physical discomfort, such as skin irritation, breathing problems or gastrointestinal problems, therefore can enhance the instrument’s content validity and sensitivity [27–29, 65]. While our study confirms the value of the GI bolt-on, further investigations are recommended to better understand the potential conceptual overlap between the GI bolt-on with pain/discomfort and to identify specific areas where a GI bolt-on could add the greatest value to the instrument.

The inclusion of the DI bolt-on resulted in a substantial improvement in explanatory power for subjective wellbeing, suggesting its capacity to capture additional information. In factor analyses, the DI bolt-on loaded onto the ‘psychosocial health’ factor alongside the CO, SL and TI bolt-ons, as well as the EQ-5D-5L anxiety/depression item, indicating potential partial overlap in constructs. However, the inclusion of more closely dining-related items might have revealed a separate ‘diet’ factor, as proposed by conceptual models for HRQoL in coeliac disease [34]. The DI bolt-on did not further enhance known-group validity after the addition of the GI bolt-on (that performed best). This may be attributed to our definition of known groups, which focused on physical health categories rather than psychosocial health or wellbeing groups. Further investigations are recommended to assess the DI bolt-on’s measurement properties in different populations, incorporating more diet-related items and questions.

This research has a few limitations. The data collection was conducted through a self-administered online survey, relying on self-reported clinical data that were not verified by physicians. The sample was not representative of the whole population of CD patients in Hungary, as all patients followed GFD at the time of the survey, and a voluntary online survey may be affected by self-selection bias. Due to the cross-sectional nature of the study, we did not evaluate test-retest reliability or responsiveness of the bolt-ons. Furthermore, the data collection took place during the second wave of Covid-19 pandemic in Hungary. This timing might have impacted the HRQoL and wellbeing of patients, potentially affecting the measurement properties of the instrument and bolt-ons. As the current study focused on assessing the added value of the bolt-ons, the determination of a final item wording was not within the scope. Future studies are recommended to better understand patients’ perspectives and experiences to finalize the item wording for the new bolt-ons. Further research is needed to investigate the psychometric properties of these bolt-ons in diverse patient groups, such as a sample consisting of both patients following and not following a GFD. Testing the bolt-ons in other relevant patient populations (e.g. gluten intolerance, irritable bowel syndrome and inflammatory bowel disease) is also recommended. Lastly, while our findings suggest the added value of bolt-ons, the impact of these bolt-ons on utilities and, consequently, their potential to enhance the sensitivity of QALY estimations in cost-utility analyses are an additional important direction for future research.

## Conclusion

Our findings suggest that the DI, GI, SL and TI bolt-ons improve the measurement performance of the EQ-5D-5L in patients with CD. Among the bolt-on items, GI demonstrated strong psychometric performance in multiple tests, suggesting its value in capturing important aspects of HRQoL, that are potentially missed by the core five dimensions of EQ-5D-5L in patients with CD.

## Electronic supplementary material

Below is the link to the electronic supplementary material.

Supplementary Material 1

## Data Availability

All data of this study are available from the corresponding author upon reasonable request.

## Code availability

N/A.

## Declarations

### JEL classification

I10.
