# Supplementary material for: The added value of the cognition, dining, gastrointestinal problems, sleep and tiredness bolt-on dimensions to the EQ-5D-5L in patients with coeliac disease
Source: Eur J Health Econ. 2024 Aug 30;26(3):473–85. doi: 10.1007/s10198-024-01719-6 (PMC11937053; doi:10.1007/s10198-024-01719-6)
Supplement: Supplementary file 1 — Supplementary Material 1 [file 10198_2024_1719_MOESM1_ESM.docx]

**Online Resource 1 The five bolt-on items tested in the study**

| **New bolt-ons** |
| --- |
| DINING (e.g. following a diet, eating out) |
| I have no problems with dining |
| I have slight problems with dining |
| I have moderate problems with dining |
| I have severe problems with dining |
| I have extreme problems with dining |
| GASTROINTESTINAL PROBLEMS (e.g. diarrhoea, constipation, nausea, vomiting, heartburn, bloating, gases) |
| I have no gastrointestinal problems |
| I have slight gastrointestinal problems |
| I have moderate gastrointestinal problems |
| I have severe gastrointestinal problems |
| I have extreme gastrointestinal problems |
| **Existing bolt-ons** |
| COGNITION (memory, comprehension, concentration, thinking) |
| I have no problems with cognition |
| I have slight problems with cognition |
| I have moderate problems with cognition |
| I have severe problems with cognition |
| I have extreme problems with cognition |
| SLEEP |
| I have no problems sleeping |
| I have slight problems sleeping |
| I have moderate problems sleeping |
| I have severe problems sleeping |
| I have extreme problems sleeping |
| TIREDNESS |
| I am not tired |
| I am slightly tired |
| I am moderately tired |
| I am severely tired |
| I am extremely tired |

***© EuroQol Research Foundation. EQ-5D™ is a trade mark of the EuroQol Research Foundation. This is a modified EQ-5D. Reproduced by permission of EuroQol Research Foundation. Reproduction of this version is not allowed. For reproduction, use or modification of the EQ-5D (any version), please register your study by using the online EQ registration page:***[***www.euroqol.org***](http://www.euroqol.org)***.***

**Online Resource 2 Pattern matrix of the principal component analysis**

| **Items** | **Factor** | | | |
| --- | --- | --- | --- | --- |
|  | **1. Gastro-intestinal problems** | **2. Satisfac-tion with life** | **3. Psycho-social health** | **4. Pain and usual activities** |
| GSRS Indigestion | 0.930 | - | - | - |
| GSRS Diarrhoea | 0.837 | - | - | - |
| GSRS Abdominal pain | 0.750 | - | - | - |
| EQ-5D-5L Gastrointestinal problems bolt-on | 0.732 | - | - | - |
| GSRS Constipation | 0.620 | - | - | - |
| GSRS Reflux | 0.600 | - | - | - |
| SWLS So far I have gotten the important things I want in life | - | 0.884 | - | - |
| SWLS I am satisfied with my life. | - | 0.877 | - | - |
| SWLS The conditions of my life are excellent. | - | 0.846 | - | - |
| SWLS In most ways my life is close to my ideal. | - | 0.824 | - | - |
| SWLS If I could live my life over, I would change almost nothing | - | 0.799 | - | - |
| EQ-5D-5L Cognition bolt-on | - | - | 0.991 | - |
| EQ-5D-5L Anxiety/depression bolt-on | - | - | 0.763 | - |
| EQ-5D-5L Sleep bolt-on | - | - | 0.606 | - |
| EQ-5D-5L Tiredness bolt-on | - | - | 0.537 | - |
| EQ-5D-5L Dining bolt-on | - | - | 0.501 | - |
| EQ-5D-5L Self-care | - | - | - | 0.919 |
| EQ-5D-5L Mobility | - | - | - | 0.854 |
| EQ-5D-5L Pain/discomfort | 0.322 | - | - | 0.476 |
| EQ-5D-5L Usual activities | - | - | 0.448 | 0.476 |

Factor loadings > 0.3 are shown.

GSRS = Gastrointestinal Symptoms Rating Scale; SWLS = Satisfaction with Life Scale
